# Universal Asymptotic Clone Size Distribution for General Population Growth

**DOI:** 10.1007/s11538-016-0221-x

**Published:** 2016-10-20

**Authors:** Michael D. Nicholson, Tibor Antal

**Affiliations:** 1 SUPA, School of Physics and Astronomy, University of Edinburgh, Edinburgh, EH9 3FD UK; 2 School of Mathematics, University of Edinburgh, Edinburgh, EH9 3FD UK

**Keywords:** Luria–Delbrück, Branching process, Clone size, Cancer

## Abstract

Deterministically growing (wild-type) populations which seed stochastically developing mutant clones have found an expanding number of applications from microbial populations to cancer. The special case of exponential wild-type population growth, usually termed the Luria–Delbrück or Lea–Coulson model, is often assumed but seldom realistic. In this article, we generalise this model to different types of wild-type population growth, with mutants evolving as a birth–death branching process. Our focus is on the size distribution of clones—that is the number of progeny of a founder mutant—which can be mapped to the total number of mutants. Exact expressions are derived for exponential, power-law and logistic population growth. Additionally, for a large class of population growth, we prove that the long-time limit of the clone size distribution has a general two-parameter form, whose tail decays as a power-law. Considering metastases in cancer as the mutant clones, upon analysing a data-set of their size distribution, we indeed find that a power-law tail is more likely than an exponential one.

## Introduction

Cancerous tumours spawning metastases, bacterial colonies developing antibiotic resistance or pathogens kickstarting the immune system are examples in which events in a primary population initiate a distinct, secondary population. Regardless of the scenario under consideration, the number of individuals in the secondary population, and how they are clustered into colonies, or *clones*, is of paramount importance. An approach which has offered insight has been to bundle the complexities of the initiation process into a mutation rate and assume that the primary, or *wild-type*, population seeding the secondary, or *mutant*, population is a random event.

This method was pioneered by microbiologist Salvador Luria and theoretical physicist Max Delbrück (Luria and Delbrück 1943). In their Nobel prize winning work, they considered an exponentially growing, virus susceptible, bacterial population. Upon reproduction, with small probability, a virus resistant mutant may arise and initiate a mutant clone. This model was contrasted with each wild-type individual developing resistance upon exposure to the virus with a constant probability per individual. By considering the variance in the total number of mutants in each case, they demonstrated that bacterial evolution developed spontaneously as opposed to adaptively in response to the environment.

In the original model of Luria and Delbrück, both wild-type and mutant populations grow deterministically, with mutant initiation events being the sole source of randomness. Lea and Coulson (1949) generalised this process by introducing stochastic mutant growth in the form of the pure birth process and were able to derive the distribution of the number of mutants for neutral mutations. This was again extended by Bartlett (1955) and later Kendall (1960), who considered both populations developing according to a birth process. An accessible review discussing these formulations is given by Zheng (1999).

Recent developments have focused on cancer modelling, where usually mutant cell death is included in the models. The main quantity of interest in these studies has been the total number of mutant cells. Explicit and approximate solutions appeared for deterministic, exponential wild-type growth, corresponding to a fixed size wild-type population (Angerer 2001; Dewanji et al. 2005; Iwasa et al. 2006; Komarova et al. 2007; Keller and Antal 2015), and fully stochastic wild-type growth either at fixed time or fixed size (Durrett and Moseley 2010; Antal and Krapivsky 2011; Kessler and Levine 2015). An exciting recent application has been to model emergence of resistance to cancer treatments (Kessler et al. 2014; Bozic et al. 2013; Bozic and Nowak 2014). The current study continues in this vein with our inspiration being primary tumours (wild-type) seeding metastases (mutant clones).

Interestingly, in the large time small mutation rate limit, the clone size distribution at a fixed wild-type population size coincides for stochastic and deterministic exponential wild-type growth (Kessler and Levine 2015; Keller and Antal 2015). The intuition behind this observation is that a supercritical birth–death branching process converges to exponential growth in the large time limit, and, for a small mutation rate, mutant clones are initiated at large times. So asymptotically the two methods are equivalent, but the deterministic description of the wild-type population has twofold advantages: (i) the calculations are much simpler in this case (Keller and Antal 2015), and (ii) the method can be easily generalised to arbitrary growth functions. This is the programme that we develop in the present paper.

The present work differs from previous approaches in two ways. Firstly, motivated by populations with environmental restrictions, we move away from the assumption of exponential wild-type growth, a setting which has received limited previous consideration as discussed in Foo and Michor (2014). We shall first review and extend results for the exponential case and then provide explicit solutions for power-law and logistic growth. Next, we present some general results which are valid for a large class of growth functions. This extends the classic results found in Kendall (1948), Athreya and Ney (2004), Karlin and Taylor (1981), Tavare (1987) and recent work in Tomasetti (2012), Houchmandzadeh (2015) who considered the wild-type population growth rate to be time-dependent but coupled with the mutant growth rate. Secondly, rather than the total number of mutants, our primary interest is on the distribution of mutant number in the clones initiated by mutation events. This complements Hanin et al. (2006), which allowed deterministic wild-type and mutant growth, and the treatment of clone sizes for constant wild-type populations found in Dewanji et al. (2011). While we focus on clone sizes, we demonstrate that the distribution for the total number of mutants follows as a consequence, and hence, results hold in that setting also.

The outline of this work is as follows. We define our model in Sect. 2, utilising formalism introduced in Karlin and Taylor (1981), and demonstrate a mapping between the mutant clone size distribution and the distribution for the total number of mutants. The exact time-dependent size distribution is given for exponential, power-law and logistic wild-type growth function in Sect. 4. Section 5 pertains to universal features of the clone size distribution and contains our most significant results. There, for a large class of wild-type growth functions, we demonstrate a general two-parameter distribution for clone sizes at large times. The distribution has power-law tail behaviour which corroborates previous work (Iwasa et al. 2006; Durrett and Moseley 2010; Williams et al. 2016). Large time results are also given for the mean and variance of the clone sizes under general wild-type growth. Adopting the interpretation of the wild-type population as the primary tumour and mutant clones as metastases, we test our results regarding the tail of the distribution on empirical metastatic data in Sect. 6. Section 7 considers alternative methods to ours, and we give some concluding remarks in Sect. 8.

## Model

**Fig. 1:**
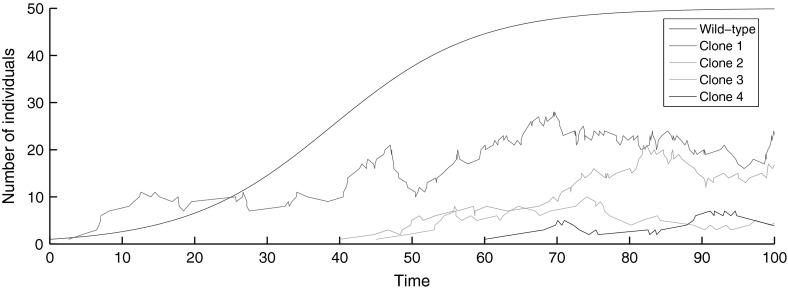
(Color figure online) A sample realisation for deterministic logistic wild-type growth, with a carrying capacity of 50, and stochastic mutant growth. Note that we typically assume the wild-type population is much larger than individual clones

In our model, a wild-type population gives rise to mutants during reproduction events. The arisen mutant also reproduces, and so mutant clones stem from the original initiating mutant’s progeny. In many applications, the wild-type population is significantly larger than the mutant clones, and so we treat the wild-type population’s growth as deterministic, with size dictated by a time-dependent function $n_{\tau }$. The mutant clones are smaller in comparison, and so their growth is stochastic. For logistic wild-type growth, a sample realisation of the process is shown in Fig. 1. The exact formulation is now given.

### The Birth–Death Process

Stochastic growth of mutants will follow a birth–death branching process (Athreya and Ney 2004). Time is scaled such that each mutant has unit birth rate and death rate $\beta$. A brief note on converting our results to the case when the birth rate is arbitrary is given in “Appendix B”. Let $Z_{t}$ be the size of a population at time *t*, with $Z_{0}=1$. The forward Kolmogorov equation for the distribution is given by

1 $$\begin{aligned} \partial _{t} {\mathbb {P}}(Z_{t}=k)=(k-1) {\mathbb {P}}(Z_{t}=k-1)+\beta (k+1) {\mathbb {P}}(Z_{t}=k+1)-(1+\beta ) k {\mathbb {P}}(Z_{t}=k) \end{aligned}$$

with $k\ge 1$. Its solution in terms of the generating function, given on page 76 of Bartlett (1955), is

2 $$\begin{aligned} {\mathcal {Z}}_{t}(s)={\mathbb {E}}(s^{Z_{t}})=1-\frac{\lambda }{1-\xi e^{-\lambda t}}, \quad \text {where}\,\xi = \frac{\beta -s}{1-s},\quad \lambda =1-\beta . \end{aligned}$$

Due to our timescale, $\beta$ is the probability of eventual extinction for a mutant clone for $\beta \le 1$, and $\lambda$ is the mutant fitness. When $\beta =0$, and so the stochastic proliferation follows a pure birth or Yule process, the mutants will be denoted immortal. By expanding the generating function around $s=0$, we obtain for the probability of the population size being *k* a geometric distribution with a modified zero term

3 $$\begin{aligned} {\mathbb {P}}(Z_{t}=k)= {\left\{ \begin{array}{ll} \beta / {\mathcal {S}}_{t} &{}\quad k=0\\ (1-\beta /{\mathcal {S}}_t) ({\mathcal {S}}_t-1)\, {\mathcal {S}}_{t}^{-k} &{}\quad k\ge 1, \end{array}\right. } \end{aligned}$$

with the shorthand notation

4 $$\begin{aligned} {\mathcal {S}}_{t}=\frac{1-\beta e^{-\lambda t}}{1-e^{-\lambda t}}. \end{aligned}$$

For the particular case of a critical branching process, i.e. when birth and death rates are equal, the above probabilities are simplified by observing

5 $$\begin{aligned} \lim _{\beta \rightarrow 1}{\mathcal {S}}_t=\frac{t+1}{t}. \end{aligned}$$

### Mutant Clone Size Distribution

Here, we employ standard methods as outlined in, for instance, Karlin and Taylor (1981), Dewanji et al. (2005). The system is observed at a fixed time *t*, and we let the number of wild-type individuals be denoted by $n_{\tau }$ for $0\le \tau \le t$. Since mutants are produced by wild-type individuals, the rate of mutant clone initiations will be proportional to the product of $n_{\tau }$ and the mutation rate $\mu$. More precisely, the process of clone initiations is an inhomogeneous Poisson process (Karlin and Taylor 1998) with intensity $\mu n_{\tau }$. Let the Poisson random variable $K_{t}$ denotes the number of clones that have been initiated by *t*, which has mean

$$\begin{aligned} {\mathbb {E}}(K_{t})=\int _{0}^{t}\mu n_{\tau }\,\hbox {d}\tau . \end{aligned}$$

Now, assuming $K_{t}>0$, we consider a mutant clone sampled uniformly from the $K_t$ initiated clones and denote its size to be the random variable $Y_{t}$. The clone was initiated at the random time *T*, and as we must have $T\le t$, the density of *T* is given by

6 $$\begin{aligned} f_{T}(\tau )=\frac{\mu n_{\tau }}{{\mathbb {E}}(K_{t})}=\frac{n_{\tau }}{a_{t}}. \end{aligned}$$

Where

7 $$\begin{aligned} a_{t}=\frac{{\mathbb {E}}(K_{t})}{\mu }=\int _{0}^{t}n_{\tau }\,\hbox {d}\tau \end{aligned}$$

is the expected number of clones seeded when the mutation rate is unity. The size of the clone is dictated not only by the initiation time but also by its manner of growth, here the birth–death process. Hence, by conditioning on the arrival time, we have

8 $$\begin{aligned} {\mathbb {P}}(Y_{t}=k)=\frac{1}{a_{t}}\int _{0}^{t} n_{\tau } {\mathbb {P}}(Z_{t-\tau }=k)\,\hbox {d}\tau . \end{aligned}$$

An immediate consequence is that the generating function of the clone size is given by

9 $$\begin{aligned} \mathcal {Y}_t(s)={\mathbb {E}}(s^{Y_{t}}) =\frac{1}{a_{t}}\int _{0}^{t} n_{\tau } {\mathcal {Z}}_{t-\tau }(s)\,\hbox {d}\tau , \end{aligned}$$

where ${\mathcal {Z}}_{t}(s)$ is the generating function of the birth–death process (2).

We make the following remarks on the above. (i) The mutation rate $\mu$ does not appear in the density for initiation times in (6); hence mutant clone sizes are independent of the mutation rate and thus all following results regarding clone sizes will be also. (ii) The integral in (8) is a convolution, and as convolutions commute, we may swap the arguments of the integrand functions ($n_{\tau }{\mathcal {Z}}_{t-\tau }\leftrightarrow n_{t-\tau }{\mathcal {Z}}_{\tau }$). (iii) If we start with $n_{0}$ wild-type individuals, so the wild-type follows $m_{\tau }=n_{0}n_{\tau }$, then both the numerator and denominator in (6) will have a factor of $n_{0}$, which cancel. So henceforth, apart from when $n_{0}=0$ (used occasionally for analytic convenience), we set $n_{0}=1$ without loss of generality. (iv) By similar logic, a positive random amplitude for the wild-type growth function, i.e. $m_{\tau }=Xn_{\tau }$ for a general positive random variable *X*, would also cancel, and so our results on clone sizes hold in that case also.

## Mapping Distributions: Clone Size to Total Mutant Number

This section is related to the classic Luria–Delbrück problem. Let $B_{t}$ be the total number of mutants existing at time *t*. Then, $B_{t}$ is the sum of $K_{t}$ generic clones

$$\begin{aligned} B_{t}=\sum _{i=1}^{K_{t}}(Y_{t})_{i}\,, \end{aligned}$$

where all $(Y_{t})_{i}$ are *iid* random variables specifying the clone sizes. As such, $B_{t}$ is a compound Poisson random variable, and hence its generating function is

10 $$\begin{aligned} \mathcal {B}_t(s)={\mathbb {E}}(s^{B_{t}})=e^{{\mathbb {E}}(K_{t})[\mathcal {Y}_t(s)-1]}, \end{aligned}$$

which can be derived by conditioning on $K_{t}$. It follows that

11 $$\begin{aligned} {\mathbb {E}}(B_{t})={\mathbb {E}}(K_{t}){\mathbb {E}}(Y_{t})\quad \text { and }\quad {\mathrm {Var}}(B_{t})={\mathbb {E}}(K_{t}){\mathbb {E}}(Y_{t}^2). \end{aligned}$$

The link between the mass functions of the mutant clone size, $Y_{t}$, and the total number of mutants, $B_{t}$, is given by the recursion

$$\begin{aligned} {\mathbb {P}}(B_{t}=n)= {\left\{ \begin{array}{ll} e^{{\mathbb {E}}(K_{t})({\mathbb {P}}(Y_{t}\,=\,\,0)-1)}&{}\quad n=0\\ {\mathbb {E}}(K_{t})\sum \limits _{k=0}^{n-1}\frac{n-k}{n}{\mathbb {P}}(B_{t}=k){\mathbb {P}}(Y_{t}=n-k) &{}\quad n\ge 1. \end{array}\right. } \end{aligned}$$

This relationship may be found as Lemma 2 in Zheng (1999), and a short proof is provided for convenience in “Appendix B”, Lemma B1. Hence, while we may initially work in the setting of size distribution of a single clone, by the above discussion, results are transferable to the total number of mutants case.

Often long-time results are sought, which significantly reduces the complexity of the distributions. For any fixed positive mutation rate, in the long-time limit, an infinite number of clones will have been initiated, and thus, the probability distributions of $B_{t}$ will not be tight (Durrett 1996). A common solution to this problem is the *Large Population-Small Mutation* limit (Keller and Antal 2015), where $\theta =\mu n_{t}$ is kept constant. Then, for exponential wild-type growth, $n_{\tau }=e^{\delta \tau },$ (or exponential-type, see Sect. 5), the expected number of initiated clones, ${\mathbb {E}}(K_t)$, tends to $\theta /\delta$ for large times. Hence, we see that

$$\begin{aligned} \begin{aligned} \lim _{\begin{array}{c} t\rightarrow \infty \\ \theta \,{\mathrm {constant}} \end{array}} \mathcal {B}_t(s)=\exp \left[ \frac{\theta }{\delta } (\lim _{t\rightarrow \infty }\mathcal {Y}_t(s)-1)\right] , \end{aligned} \end{aligned}$$

demonstrating that the limit of the clone size distribution is of primary concern. Furthermore, if the expected number of initiated clones is small, we have the following proposition, whose proof can be found in “Appendix B”.

### Proposition 1

For a small expected number of initiated clones, conditioned on survival, the size of a single clone and the total number of mutants are approximately equal in distribution. That is,

$$\begin{aligned} {\mathbb {P}}(B_{t}=k|B_{t}>0) = {\mathbb {P}}(Y_{t}=k|Y_{t}>0)+O({\mathbb {E}}(K_{t}) ),\quad \text {as}\quad {\mathbb {E}}(K_{t})\rightarrow 0. \end{aligned}$$

One immediate consequence of this result is that for immortal mutants ($\beta =0$) and ${\mathbb {E}}(K_{t}) \ll 1$ we have

$$\begin{aligned} \mathcal {B}_t(s)\approx (1-e^{-{\mathbb {E}}(K_{t}) })\mathcal {Y}_t(s)+e^{-{\mathbb {E}}(K_{t}) }\implies {\mathbb {P}}(B_{t}=k)\approx {\mathbb {E}}(K_{t}) {\mathbb {P}}(Y_{t}=k) \quad \text {for}\, k\ge 1. \end{aligned}$$

This agrees with intuition as for small enough ${\mathbb {E}}(K_{t})$, we expect only 0 or 1 clones to be initiated, and hence, the total number of mutants will be dictated by the clone size distribution. With exponential wild-type growth, this approximation was used in Iwasa et al. (2006) to investigate drug resistance in cancer.

## Finite Time Clone Size Distributions

**Fig. 2:**
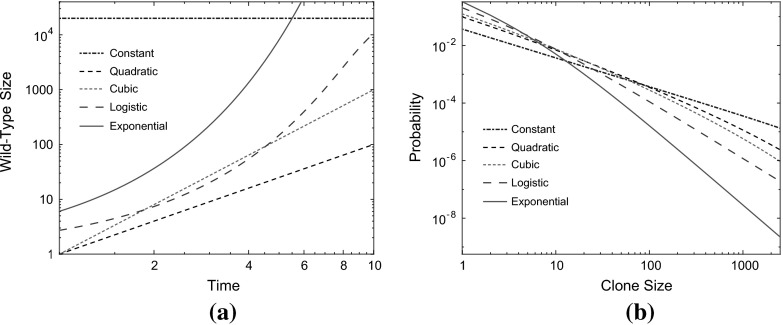
**a** Growth curves for different wild-type growth functions $n_{\tau }$. **b** The associated probability mass functions, derived in Sect. 4, for the clone size when wild-type follows growth curves shown in **a**. Parameters: $\delta =1.8, \,\lambda =1,\,t=9, K=20{,}000$

Three particular cases of wild-type growth function, $n_{\tau }$, will be considered in detail, namely: exponential, power-law and logistic (Fig. 2). Exponential and logistic growth are widely used in biological modelling (Murray 2002). For the power-law cases, under the assumption that the radius of a spherical wild-type population is proportional to time, quadratic and cubic power-law growth represents mutation rates proportional to the surface area and volume, respectively. In each case, we give the generating function and probability mass function. We stress again that the mutation rate and an arbitrary positive prefactor for $n_{\tau }$ cancel in (8) and so are irrelevant for our results.

### Exponential Wild-Type Growth

Let the wild-type population grow exponentially, that is $n_{\tau }=e^{\delta \tau }$ with $\delta >0$ and so from (7), $a_{t}=\frac{e^{\delta t}-1}{\delta }$. The distribution for the total number of mutants, $B_{t}$, was treated exhaustively in Keller and Antal (2015), and we follow their notation by letting $\gamma =\delta /\lambda$. Using (10) and the results found in section 3 of Keller and Antal (2015), the generating function is

12 $$\begin{aligned} \mathcal {Y}_t(s)=1+\frac{\lambda }{1-n_{t}^{-1}}\bigg [ n_t^{-1} \mathop {F}\nolimits \!\left( \genfrac{}{}{0.0pt}0{1,\gamma }{1+\gamma };\xi n_{t}^{-1/\gamma }\right) -\mathop {F}\nolimits \!\left( \genfrac{}{}{0.0pt}0{1,\gamma }{1+\gamma };\xi \right) \bigg ]. \end{aligned}$$

Similarly, the mass function is

$$\begin{aligned} {\mathbb {P}}(Y_{t}=0)=1+\frac{\lambda }{1-n_{t}^{-1}}\bigg [n_t^{-1} \mathop {F}\nolimits \!\left( \genfrac{}{}{0.0pt}0{1,\gamma }{1+\gamma };\beta n_{t}^{-1/\gamma }\right) -\mathop {F}\nolimits \!\left( \genfrac{}{}{0.0pt}0{1,\gamma }{1+\gamma };\beta \right) \bigg ] \end{aligned}$$

and for $$k\ge 1$$

$$\begin{aligned} \begin{aligned} {\mathbb {P}}(Y_{t}=k)&= \frac{\delta }{( n_{t}-1)}\sum _{j=1}^{k} {k-1\atopwithdelims ()j-1}\frac{1}{j+\gamma }\bigg (\frac{\lambda }{\beta -n_{t}^{1/\gamma }}\bigg )^{j}\mathop {F}\nolimits \!\left( \genfrac{}{}{0.0pt}0{1,\gamma }{1+\gamma +j};\beta n_{t}^{-1/\gamma }\right) \\&\quad +\,\frac{\delta }{(1-n_{t}^{-1})}\frac{(k-1)!}{(\gamma +1)_{k}}\mathop {F}\nolimits \!\left( \genfrac{}{}{0.0pt}0{k,\gamma }{1+\gamma +k};\beta \right) . \end{aligned} \end{aligned}$$

Here, $\mathop {F}\nolimits \!\left( \genfrac{}{}{0.0pt}0{a,b}{c};z\right)$ is Gauss’s hypergeometric function, and $(a)_{k}$ is the Pochhammer symbol defined in “Appendix A”. The above expressions are given in terms of $n_{t}$ to allow easy comparison to the formulas in Keller and Antal (2015). For these exact time-dependent formulas, their form is somewhat cumbersome; however, simpler long-time limit expressions are given in Sect. 5. A reduction in complexity is also obtained for the special case of neutral mutants ($\delta =\lambda$) where, by using (24), the generating function in (12) simplifies to

$$\begin{aligned} \mathcal {Y}_t(s) = 1+ \frac{\lambda }{\xi (1-e^{-\delta t})} \log \frac{1-\xi }{1-\xi e^{-\delta t}}. \end{aligned}$$

If additionally the neutral mutants are immortal, the above expression further simplifies to

$$\begin{aligned} \mathcal {Y}_t(s) = 1+ \frac{1-s}{s\phi } \log (1-s\phi )\quad \text {where}\,\phi = 1-e^{-\delta t}. \end{aligned}$$

The probabilities are then concisely given by

$$\begin{aligned} {\mathbb {P}}(Y_t=k) = \frac{\phi ^{k-1}}{k} - \frac{\phi ^k}{k+1} \quad \text{ or }\quad {\mathbb {P}}(Y_t>k)=\frac{\phi ^{k}}{k+1} \end{aligned}$$

which corresponds to the classical Lea–Coulson result (Lea and Coulson 1949)

$$\begin{aligned} \mathcal {B}_t(s) = (1-s\phi )^{\theta (1-s)/s} \end{aligned}$$

with $$\theta =\mu e^{\delta t}$$.

### Power-Law Wild-Type Growth

Now, we assume that the wild-type population grows according to a general power-law $n_{\tau }=\tau ^{\rho }$, for some non-negative integer $\rho$, and therefore, $a_{t}=\frac{t^{\rho +1}}{\rho +1}$. With power-law wild-type growth and stochastic mutant proliferation, the mutant clone size generating function is given by

13 $$\begin{aligned} \mathcal {Y}_t(s)=\beta +\lambda (\rho +1) !\bigg [\frac{(-1)^{\rho } {\mathrm {Li}}_{\rho +1}( \xi e^{-\lambda t})}{(t\lambda )^{\rho +1}}+\sum _{i=0}^{\rho }\frac{(-1)^{i+1} {\mathrm {Li}}_{i+1}(\xi )}{(\rho -i)! (t\lambda )^{i+1}} \bigg ]. \end{aligned}$$

Here, ${\mathrm {Li}}_{i}(s)$ is the polylogarithm of order *i* defined in “Appendix A”. Details of the derivation are given in “Appendix C”. For immortal mutants, the mass function may be explicitly written as

14 $$\begin{aligned} {\mathbb {P}}(Y_{t}=m)&=\frac{(\rho +1)}{m t }+\frac{(\rho +1)!}{mt}\bigg [\frac{(-1)^{\rho }}{(t)^{\rho }}\sum _{k=1}^{m}{m\atopwithdelims ()k}\frac{(-e^{-t})^{k}}{k^{\rho }} \nonumber \\&\quad +\sum _{i=1}^{\rho }\frac{(-1)^{i+1}}{(t)^{i}(\rho -i)!}\sum _{k=1}^{m}{m\atopwithdelims ()k}\frac{(-1)^{k}}{k^{i}} \bigg ]. \end{aligned}$$

If mutants may die, the exact mass function is most easily obtained via Cauchy’s integral formula which may be efficiently computed using the fast Fourier transform. For a brief discussion on implementation, see Antal and Krapivsky (2010) and references therein.

Note for $\rho \ge 1$, $n_{0}=0$ which, while useful for analytic tractability, is unrealistic. This can be overcome by letting $n_{\tau }=n_{0}+\tau ^{\rho }$. Then, by splitting the integral in the generating function (9) and using the above analysis, one can obtain the mass function for any $n_{0}$. However, for practical purposes, the contribution of $n_{0}$ is negligible.

### Constant Size Wild-Type

For the specific power-law growth when $\rho =0$, i.e. $n_{\tau }=1$ (recall that this is equal to the general case when $n_{\tau }=n_{0}$), we recover some classical results for constant immigration (Kendall 1948). We note that the distribution of the ordered clone size, depending on initiation time, was discussed in Jeon et al. (2008). From (13) with $\rho =0$, the generating function is

15 $$\begin{aligned} \mathcal {Y}_t(s)=1-\frac{1}{t}\log \left( \frac{1-s{\mathcal {S}}_t^{-1}}{1-{\mathcal {S}}_t^{-1}} \right) . \end{aligned}$$

with ${\mathcal {S}}_t$ as given in (4). By expanding this generating function in terms of *s* we obtain the probabilities

16 $$\begin{aligned} {\mathbb {P}}(Y_{t}=k)= {\left\{ \begin{array}{ll} 1+t^{-1}\log (1-{\mathcal {S}}_t^{-1}) &{}\quad k=0\\ \frac{1}{tk}\,{\mathcal {S}}_t^{-k} &{}\quad k\ge 1. \end{array}\right. } \end{aligned}$$

Then, using (10) with the clone sizes (15) we obtain the generating function of the total number of mutants

$$\begin{aligned} \mathcal {B}_t(s) = \left[ \frac{1-{\mathcal {S}}_{t}^{-1}}{1-s{\mathcal {S}}_{t}^{-1}} \right] ^{\mu }, \end{aligned}$$

and from the binomial theorem we also get the probabilities

$$\begin{aligned} {\mathbb {P}}(B_t=m) = \left( {\begin{array}{c}m+\mu -1\\ m\end{array}}\right) \left( 1-{\mathcal {S}}_{t}^{-1}\right) ^{\mu } {\mathcal {S}}_t^{-m}. \end{aligned}$$

We recognise this as a negative binomial distribution under the interpretation that $B_{t}$ is the number of failures until $\mu$ successes, with failure probability ${\mathcal {S}}_{t}^{-1}$. This result for $B_{t}$ was first derived by Kendall (1948) who was attempting to explain the appearance of the logarithmic distribution for species number when randomly sampling heterogeneous populations, conjectured by R.A. Fisher. From the distribution of $B_{t}$, by an argument which may be considered a special case of Proposition 1, he derived that for constant rate initiation, the clone size conditioned on non-extinction is logarithmically distributed again with parameter ${\mathcal {S}}_{t}^{-1}$, which can be obtained via (16).

Constant immigration may imply a constant size source; hence, mutants with equal birth and death rates (i.e. evolving as a critical branching process) are particularly interesting. This case yields analogous formulas to those above but ${\mathcal {S}}_t$ is replaced with the expression given in (5).

### Logistic Wild-Type Growth

Starting from a population of one and having a carrying capacity *K*, logistic growth is given by $n_{\tau }=\frac{K e^{\lambda \tau }}{K+e^{\lambda \tau }-1}$. We assume neutral mutations, i.e. $\lambda$ is also the wild-type growth rate. Integrating the growth function gives $a_{t}=\frac{K}{\lambda }\log \big (\frac{e^{\lambda t}}{n_{t}}\big ).$

We aim to calculate the generating function using (9). Recalling the definition of ${\mathcal {Z}}_{t-\tau }(s)$ we observe that

$$\begin{aligned} \int \frac{1}{1-\xi e^{-\lambda (t-\tau )}}n_{\tau }\,\hbox {d}\tau =\frac{K}{\lambda [(K-1)\xi e^{-\lambda t}+1]}\log \bigg (\frac{1-e^{\lambda \tau }-K}{1-Ae^{\lambda \tau }}\bigg )+C, \end{aligned}$$

where *C* is an integration constant. Therefore, the generating function is

$$\begin{aligned} \mathcal {Y}_t(s)=1+\frac{\lambda e^{\lambda t}}{[e^{\lambda t}+(K-1)\xi ]\log (\frac{e^{\lambda t}}{n_{t}})}\log \bigg (\frac{n_{t}(1-\xi )}{e^{\lambda t}(1-\xi e^{-\lambda t})}\bigg ). \end{aligned}$$

Agreeing with intuition for $K=1$, we recover the generating function of the constant case, and $\lim _{K\rightarrow \infty }\mathcal {Y}_t(s)$ gives the generating function for exponential wild-type growth. Therefore, the logistic case interpolates between the constant and exponential growth cases. The mass function can be obtained by expanding the non-logarithmic and logarithmic function in $\mathcal {Y}_t(s)$ and using the Cauchy product formula. However, this method provides little insight, and numerically, it is simpler to use the fast Fourier transform.

### Monotone Distribution and Finite Time Cut-Off

We conclude this section by demonstrating general features that exist in the clone size distribution at finite times. Again proofs are provided in “Appendix C”. Firstly, we see that, regardless of the particular wild-type growth function, the monotone decreasing nature of the mass function for the birth–death process is preserved in the clone size distribution.

#### Proposition 2

As long as $n_{\tau }$ is positive for some subinterval of [0, *t*], then for $k\ge 1$ we have ${\mathbb {P}}(Y_{t}=k+1)< {\mathbb {P}}(Y_{t}=k)$ for any finite $t>0$.

Whether ${\mathbb {P}}(Y_{t}=0)\ge {\mathbb {P}}(Y_{t}=1)$ depends on $n_{\tau }$ and *t*, but the inequality is typically true for long times. Note that in contrast, the mass function of the total number of mutants is not monotone in general (Keller and Antal 2015).

Now restricting ourselves to the $\lambda >0$ case, as an example, consider the mass function when the size of the wild-type population is constant, which is given by (16), and specifically for $k\ge 1$. For any moderate *t*, ${\mathcal {S}}_{t}^{-1}$ is typically close to unity but for large *k*, ${\mathcal {S}}_{t}^{-k}$ will become the dominant term in the mass function, dictating exponential decay. We term this a cut-off in the distribution which occurs at approximately $k= O (e^{\lambda t})$. It is an artefact of the mass function for the birth–death process (3). Hence, we will have (at least) two behaviour regimes for the mass function for finite times. Here, we show that this cut-off exists generally for finite times.

#### Theorem 1

Let $\lambda >0$ and $n_{\tau }$ be continuous and positive for $\tau \in [0,t]$. Then

$$\begin{aligned} {\mathbb {P}}(Y_{t}=k)={\mathcal {S}}_{t}^{-k}\varTheta _t(k), \end{aligned}$$

where $\varTheta _t(k)$ is an unspecified subexponential factor, i.e. $\limsup _{k\rightarrow \infty }\root k \of {\varTheta _t(k)}=1$, and ${\mathcal {S}}_t$ is given by (4).

Note that ${\mathcal {S}}_{t}>1$ for finite *t*, and ${\mathcal {S}}_{t}\rightarrow 1$ exponentially fast for large *t*. Hence, the cut-off will disappear for long times and the subexponential factor, discussed in detail in Sect. 5, will completely determine the tail of the distribution. Also notice that the power-law cases, $n_{\tau }=\tau ^{\rho }$, for $\rho \ge 1$ are not covered as, to make the analysis tractable, they artificially start at $n_{0}=0$. However, the generating function in this case (13) also has its closest to origin singularity at ${\mathcal {S}}_{t}$ so the cut-off exists there also.

## Universal Large Time Features

Here, we give results regarding the large time behaviour of our model which is relevant in many applications and also provides general insight. In many applications, the cut-off location ($k=O(e^{\lambda t}$)) is so large that the distribution at or above this point is of little relevance, and hence, for this purpose the limiting approximations now discussed are of particular interest. Using the notation of Theorem 1, this section investigates the large time form of $\varTheta _{t}(k)$. The proofs for the results presented in this section can be found in “Appendix D”. We highlight the power-law decaying, “fat” tail found in each case. Henceforth, we again assume $\lambda >0$, i.e. a supercritical birth–death process.

### General Wild-Type Growth Functions

To give general results, we introduce the following assumption which will be assumed to hold for the remainder of this section.

#### Assumption 1

For wild-type growth function $n_{\tau }$, we assume

(i) $n_{\tau }=0$ for $\tau <0$, continuous for $\tau > 0$ and right continuous at $\tau =0$.
(ii) $n_{\tau }$ is positive and monotone increasing for $\tau > 0$.
(iii) For $x\ge 0$ the limit $\lim _{t\rightarrow \infty }n_{t-x}/n_{t}$ exists, is positive and finite.

We note that the cases discussed in Sect. 4 are all covered by Assumption 1. The reason for the seemingly arbitrary limit assumed in (iii) becomes clear with the following result which is an application of the theory of regular variation found in Bingham et al. (1987).

#### Lemma 1

For $$x\ge 0$$

$$\begin{aligned} \lim _{t\rightarrow \infty }\frac{n_{t-x}}{n_{t}}=e^{-x\delta ^*},\text { where }\lim _{t\rightarrow \infty }\frac{\log n_{t}}{t}=\delta ^*\ge 0. \end{aligned}$$

Often the long-time behaviour of the clone size distribution may be separated into $\delta ^*>0$ and $\delta ^*=0$, and so we give the following definition (Flajolet and Sedgewick 2009).

#### Definition 1

Consider a real valued function *f*(*x*) such that

$$\begin{aligned} \lim _{x\rightarrow \infty }\frac{\log f(x)}{x}=\delta ^* \end{aligned}$$

holds for some $\delta ^*\in {\mathbb {R}}$. Then, *f*(*x*) is of *exponential-type* for $\delta ^*\ne 0$ and is *subexponential* for $\delta ^*=0$.

Simple examples of subexponential functions are $e^{\sqrt{t}},\, \log (t)$, $t^{\rho }$, while $e^{\delta t}$, $e^{\delta t}t^{\rho }$ are of exponential-type, with $\delta ,\rho \in {\mathbb {R}}$.

### Mean and Variance

We now address the asymptotic properties of the clone size distribution by first discussing its mean and variance.

#### Theorem 2

With $s_{i}(t)$ subexponential functions such that $s_{1}(t),\,s_{3}(t)\rightarrow \infty$

$$\begin{aligned} {\mathbb {E}}(Y_{t})\sim {\left\{ \begin{array}{ll} \frac{\delta ^*}{\delta ^*-\lambda } &{}\quad \lambda<\delta ^*\\ s_{1}(t) &{}\quad \delta ^*=\lambda \\ e^{(\lambda -\delta ^*) t}s_{2}(t)&{}\quad \delta ^*<\lambda \end{array}\right. } \quad {\mathrm {Var}}(Y_{t})\sim {\left\{ \begin{array}{ll} \frac{\delta ^*}{\lambda }\left( \frac{2}{\delta ^*-2\lambda }-\frac{2-\lambda }{\delta ^*-\lambda }\right) -\left( \frac{\delta ^*}{\delta ^*-\lambda }\right) ^2 &{}\quad 2\lambda<\delta ^*\\ s_{3}(t) &{}\quad \delta ^*=2\lambda \\ e^{(2\lambda -\delta ^*)t}s_4(t) &{}\quad \delta ^*<2\lambda \end{array}\right. } \end{aligned}$$

as $$t\rightarrow \infty $$.

**Fig. 3:**
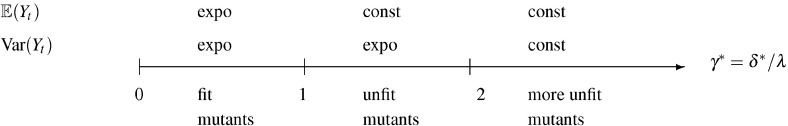
Illustration of the asymptotic behaviour of the mean and variance as given in Theorem 2

The leading asymptotic behaviour which has different regimes dependent on $\delta ^*/\lambda$ is illustrated in Fig. 3. As an example, for the exponential case $n_{\tau }=e^{\delta \tau }$, by using (11) and the results found in Keller and Antal (2015), then $\delta ^*=\delta ,\,s_{1}(t)=\lambda t,\,s_{2}(t)=\frac{\delta }{\lambda -\delta },\,s_{3}(t)=4 t$ and $s_{4}(t)=\frac{2\delta }{\lambda (2\lambda -\delta )}$.

### Large Time Clone Size Distribution

Turning to the distribution function, we have the following result regarding the generating function at large times.

#### Theorem 3

Let $\gamma ^*= \delta ^*/\lambda$. Then for $|s|<1$

$$\begin{aligned} \lim _{t\rightarrow \infty }\frac{a_{t}}{n_{t}}(\mathcal {Y}_t(s)-\beta )=\frac{1}{\gamma ^*}\left[ 1-\mathop {F}\nolimits \!\left( \genfrac{}{}{0.0pt}0{1,\gamma ^*}{1+\gamma *};\xi \right) \right] =-\sum _{k\ge 1}\frac{\xi ^{k}}{\gamma ^*+k}. \end{aligned}$$

This result is made clearer in the next corollary, in which the cases of exponential-type and subexponential growth are separated. This is as, for $\delta ^*>0$,

$$\begin{aligned} \lim _{t\rightarrow \infty }\frac{n_{t}}{a_{t}}\rightarrow \delta ^* . \end{aligned}$$

For a proof, see Lemma D1. Consequently, in the exponential-type setting, the limiting result is a proper probability distribution, while in the subexponential case it is not. We can interpret this as the clone sizes staying finite in the exponential case but grow to infinity for subexponential cases at large times. Henceforth, for brevity, we do not impose such a separation but the reader should note that for exponential-type growth the above limit holds and may simplify further results.

#### Corollary 1

For $$|s|<1$$,

$$\begin{aligned}&\lim _{t\rightarrow \infty }(\mathcal {Y}_t(s)-\beta )=\lambda \left[ 1-\mathop {F}\nolimits \!\left( \genfrac{}{}{0.0pt}0{1,\gamma ^*}{1+\gamma *};\xi \right) \right] \quad&\gamma ^*>0,\\&\lim _{t\rightarrow \infty }\frac{a_{t}}{n_{t}}(\mathcal {Y}_t(s)-\beta )=\log (1-\xi ) \quad&\gamma ^*=0, \end{aligned}$$

where the second expression is the $\gamma ^*\rightarrow 0$ limit of the first expression. Then for $t\rightarrow \infty$ the probabilities for exponential-type growth $\gamma ^*>0$ are

$$\begin{aligned} {\mathbb {P}}(Y_{t}=k)\sim {\left\{ \begin{array}{ll} 1-\lambda \mathop {F}\nolimits \!\left( \genfrac{}{}{0.0pt}0{1,\gamma ^*}{1+\gamma ^*};\beta \right) &{}\quad k=0\\ \frac{\delta ^*\varGamma (k)}{(\gamma ^*+1)_{k}}\mathop {F}\nolimits \!\left( \genfrac{}{}{0.0pt}0{k,\gamma ^*}{1+\gamma ^*+k};\beta \right) &{}\quad k\ge 1, \end{array}\right. } \end{aligned}$$

and for subexponential growth ($\gamma ^*=0$)

$$\begin{aligned} {\mathbb {P}}(Y_{t}=k)\sim {\left\{ \begin{array}{ll} \beta +\frac{n_{t}\log (\lambda )}{a_{t}} &{}\quad k=0\\ \frac{n_{t}}{a_{t} k} &{}\quad k\ge 1. \end{array}\right. } \end{aligned}$$

**Fig. 4:**
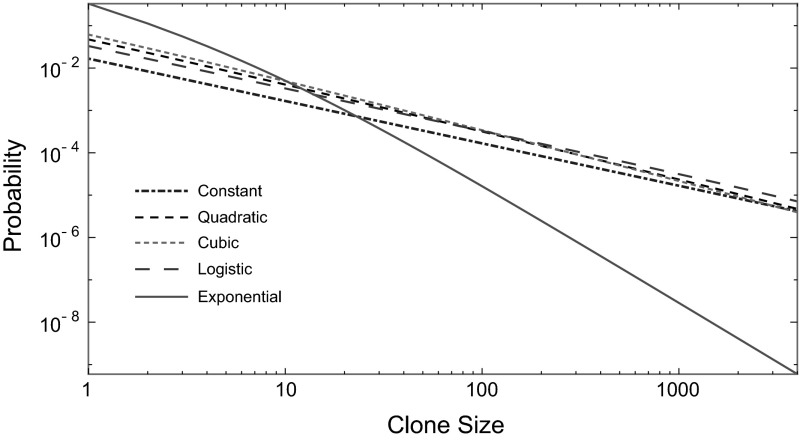
Transition to the asymptotic regime as described in Corollary 1. For subexponential wild-type growth, the mass functions tend to $k^{-1}$ behaviour, while for exponential-type it tends to $k^{-1-\gamma ^*}$. Here, $t=20$ and all other parameters are as given in Fig. 2

This result is exemplified in Fig. 4. The expressions obtained in the $\delta ^*>0$ case also appeared as an approximation in Kessler and Levine (2015) for the total number of mutants with stochastic wild-type and mutant growth when the mean number of clones is small. This can now be interpreted as an application of Proposition 1.

The case of immortal mutants does not simplify the above expressions for subexponential growth, but for exponential-type growth, by applying (23) then (22) to the limiting generating function, we have the following link to the Yule-Simon distribution which appears often in random networks (Simon 1955; Krapivsky and Redner 2001).

#### Corollary 2

For immortal mutants with exponential-type wild-type growth the clone size distribution $Y_{t}$ follows a Yule-Simon distribution with parameter $\delta ^*$ for large times. That is, for $\beta =0,\,\delta ^*>0$,

$$\begin{aligned} \lim _{t\rightarrow \infty }\mathcal {Y}_t(s)=\frac{s\delta ^*}{\delta ^*+1}\mathop {F}\nolimits \!\left( \genfrac{}{}{0.0pt}0{1,1}{2+{\delta ^*}};s\right) , \end{aligned}$$

and thus, for $k\ge 1$,

$$\begin{aligned} \lim _{t\rightarrow \infty }{\mathbb {P}}(Y_{t}=k)=\frac{\delta ^*\varGamma (k)}{(\delta ^*+1)_{k}} . \end{aligned}$$

With immortal, neutral ($\delta ^*=1$) mutants we have

$$\begin{aligned} \lim _{t\rightarrow \infty } {\mathbb {P}}(Y_t=k) = \frac{1}{ k(k+1)}. \end{aligned}$$

which is in agreement with the long-time limit of (4.1). For immortal mutants and exponential-type growth, as the clone size distribution tends to a Yule-Simon distribution, we expect power-law tail behaviour at large times (Newman 2005). Interestingly, we see that this behaviour holds when we include mutant death and have general wild-type growth.

#### Corollary 3

At large times, the tail of the clone size distribution follows a power-law with index $1+\gamma ^*$. More precisely,

$$\begin{aligned} \lim _{k\rightarrow \infty }\lim _{t\rightarrow \infty }\frac{k^{\gamma ^*+1}a_{t}}{n_{t}}{\mathbb {P}}(Y_{t}=k)=\frac{\varGamma (1+\gamma ^*) }{\lambda ^{\gamma ^*}}. \end{aligned}$$

### Large Time Distribution for Total Number of Mutants

Finally, to conclude this section, we give the corresponding results for the total number of mutants $B_{t}$ in the often used *Large Population-Small Mutation* limit.

#### Theorem 4

Letting $\theta =\mu n_{t}$ be constant and with $s_{i}(t)$ subexponential functions as in Theorem 2

$$\begin{aligned} {\mathbb {E}}(B_{t})\sim {\left\{ \begin{array}{ll} \frac{\theta a_{t}}{n_{t}} \frac{\delta ^*}{\delta ^*-\lambda } &{}\quad \lambda<\delta ^*\\ \frac{\theta a_{t}}{n_{t}} s_{1}(t) &{}\quad \delta ^*=\lambda \\ \frac{\theta a_{t}}{n_{t}}e^{(\lambda -\delta ^*) t}s_{2}(t)&{}\quad \delta ^*<\lambda \end{array}\right. } \quad {\mathrm {Var}}(B_{t})\sim {\left\{ \begin{array}{ll} \frac{\theta a_{t}}{n_{t}} \frac{\delta ^*}{\lambda }\left( \frac{2}{\delta ^*-2\lambda }-\frac{2-\lambda }{\delta ^*-\lambda }\right) &{}\quad 2\lambda<\delta ^*\\ \frac{\theta a_{t}}{n_{t}} s_{3}(t) &{}\quad \delta ^*=2\lambda \\ \frac{\theta a_{t}}{n_{t}}e^{(2\lambda -\delta ^*)t}s_4(t) &{}\quad \delta ^*<2\lambda \end{array}\right. } \end{aligned}$$

as $$t\rightarrow \infty $$. For $$|s|<1$$

$$\begin{aligned} \lim _{\begin{array}{c} t\rightarrow \infty \\ \theta \,{\mathrm { constant}} \end{array}}\mathcal {B}_t(s)\exp \left( \frac{\theta a_{t}\lambda }{n_{t}}\right) =\exp \left( \frac{\theta }{\gamma ^*}\left[ 1-\mathop {F}\nolimits \!\left( \genfrac{}{}{0.0pt}0{1,\gamma ^*}{1+\gamma *};\xi \right) \right] \right) , \end{aligned}$$

and we have the following tail result

$$\begin{aligned} \lim _{k\rightarrow \infty }\lim _{\begin{array}{c} t\rightarrow \infty \\ \theta \,{\mathrm { constant}} \end{array}}k^{\gamma ^*+1}\exp \left( \frac{\theta a_{t}}{n_{t}}\right) {\mathbb {P}}(B_{t}=k)=\frac{\theta \varGamma (1+\gamma ^*) }{\lambda ^{\gamma ^*}}. \end{aligned}$$

## Tail Behaviour in Empirical Metastatic Data

Given the above discussion we expect, for a large class of wild-type growth functions, to see power tail behaviour on approach to the exponential cut-off in the clone size distribution. We take the first steps to verify this theoretical hypothesis by analysing an empirical metastatic data. In this setting, the wild-type population is the primary tumour and mutant clones are the metastases.

Our data are sourced from the supplementary materials in Bozic et al. (2013). These data are taken from 22 patients; 7 with pancreatic ductal adenocarcinomas, 11 with colorectal carcinomas, and 6 with melanomas. One patient had only a single metastasis so we discard this data. Of the 21 remaining patients, the number of cells in a single metastasis ranged from $6\times 10^6$ to $2.23\times 10^9$. Our theoretical model predicts a cut-off in the distribution around $k=e^{\lambda t}$. Taking some sample parameters from the literature, namely $\lambda =0.069$/day (Diaz et al. 2012), and $t=14.1$ years (Yachida et al. 2010), this leads to a cut-off around $k\approx 10^{154}$ cells. Due to the enormity of this value, we ignore the cut-off here. Additionally, as the minimum observed metastasis size is $6\times 10^6$ cells, we assume that all data points are sampled from the tail of the distribution.

For each of the data-sets, we examine the likelihood ratio to determine whether the data is more likely sampled from a power-law decaying or geometrically decaying distribution. Nineteen of the 21 data-set return the power-law hypothesis as more plausible which is in agreement with the theoretical prediction. Both are single parameter distributions, and maximum likelihood analysis was utilised to estimate the parameters. The methodology outlined in Clauset et al. (2009) was broadly followed, and brief details regarding calculating maximum likelihood estimates (MLEs) are given in “Appendix E”. We note that in this context the likelihood ratio point esimator returns equivalent results to the Akaike information criterion widely used in model selection (Burnham and Anderson 1998). Under the power-law model, ${\mathbb {P}}(Y_{t}=k)\propto k^{-\omega }$, for 20 of the 21 data-sets, we find the point estimate of the power-law index, $\hat{\omega }$, lies in $[-2,-1]$. The outlier comes from the smallest data-set (3 metastases). Due to the small size of data-sets, we recognise the influence of statistical fluctuations.

**Fig. 5:**
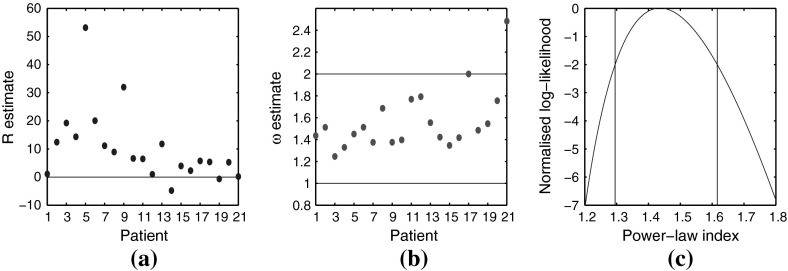
Likelihood analysis results: patients are sorted left to right by number of metastases with patient 1 having 30 mets to patient 21 having 3. Hence, values to left of figures are more significant. **a** Likelihood ratio $\hat{\mathcal {R}}$ for each data-set. Points above the horizontal line suggest data-set is from a power-law distribution over a geometric distribution. **b** Estimate $\hat{\omega }$ for each data-set, determined via maximum likelihood. **c** Normalised log-likelihood function for best data-set. Vertical bars show the likelihood interval

Details of the likelihood ratio are as follows. Let $\mathbf y =(y_{1},\ldots ,y_{N})$ be a data-set of size *N*. We test the hypothesis that $\mathbf y$ is drawn from a power-law distribution, ${\mathbb {P}}_{1}(Y_{t}=k)=C_{1}k^{-\omega }$, versus that it is sampled from a geometric distribution, ${\mathbb {P}}_{2}(Y_{t}=k)=C_{2}p(1-p)^{k}$, where $C_{1},\,C_{2}$ are normalising constants and *p* is the parameter for the geometric distribution. The log-likelihood ratio is

$$\begin{aligned} \hat{\mathcal {R}}=\sum _{i=1}^{N}[\log {\mathbb {P}}_{1}(Y_{t}=y_{i})-\log {\mathbb {P}}_{2}(Y_{t}=y_{i})], \end{aligned}$$

where $\hat{\mathcal {R}}>0$ gives support to the hypothesis that the data is drawn from the power-law distribution with MLE exponent $\hat{\omega }$, over the geometric distribution with MLE parameter $\hat{p}$. The results are given in Fig. 5a.

Assuming a power-law distribution, the maximum likelihood estimates for the exponent $\omega$ for each data-set are given in Fig. 5b. Due to the small sample size of our data-sets and the high variance in the distribution, we do not derive confidence intervals via normal distribution approximations. Instead we show the normalised log-likelihood, $\log \mathfrak {L}(\omega )/\mathfrak {L}(\hat{\omega })$, for our best data-set, with $N=30$, in Fig. 5c, where $\mathfrak {L}(\omega )$ is the likelihood function. Also, following Hudson (1971), we demonstrate the likelihood interval defined as

$$\begin{aligned} I(\omega )=\left\{ \omega :\log \frac{\mathfrak {L}(\omega )}{\mathfrak {L}(\hat{\omega })}\ge -2\right\} . \end{aligned}$$

If a large sample size was possible this interval would correspond to a $95.4\%$ confidence interval. For the data-set with $N=30$, we numerically determined $I(\omega )=[1.295,1.616]$, demonstrated as the domain between the vertical bars in Fig. 5c.

## Alternative Approaches

### Deterministic Approximation

In order to circumvent the complexity introduced by the birth–death process, one might be tempted to simply assume the mutant clone size grows according to $e^{\lambda \tau }$, the mean of the birth–death process. This approach corroborates our results regarding the tail of the size distribution. Indeed, the clone size density may be found to be

17 $$\begin{aligned} f_{Y_t}(y)=\frac{n_{t-\frac{\log (y)}{\lambda }}}{a_{t}\lambda y}. \end{aligned}$$

which has support $[1,e^{\lambda t}]$. This formula can also be found in Hanin et al. (2006). Then, as in Sect. 5 under Assumption 1,

$$\begin{aligned} \lim _{t\rightarrow \infty }\frac{a_{t}}{n_{t}}f_{Y_t}(y)=\frac{1}{y^{\gamma ^*+1}\lambda }. \end{aligned}$$

Thus, asymptotically the density has the same behaviour as the tail of the limiting result given in Corollary 3, but with a different amplitude.

However, despite this agreement, the densities given by (17) for specific wild-type growth function differ significantly compared with stochastic mutant proliferation. Letting $Y_{t}^{\mathrm {Stoch}}$ be the clone size distribution with stochastic mutant growth and $Y_{t}^{\mathrm {Det}}$ be its deterministic approximation specified by (17), we may quantify the approximation error, at least for the moments, by the following theorem, whose proof can be found in “Appendix F”.

#### Theorem 5

As $$t\rightarrow \infty $$

$$\begin{aligned} \frac{{\mathbb {E}}[(Y_{t}^{\mathrm {Stoch}})^{m}]}{{\mathbb {E}}[(Y_{t}^{\mathrm {Det}})^{m}]}=\frac{m!}{\lambda ^{m-1}}+O(e^{-\lambda t}). \end{aligned}$$

### Time-Dependent Rate Parameters

Some authors Houchmandzadeh (2015), Tomasetti (2012) have previously considered the case where all rates in the system are multiplied by a time-dependent function, say $z(\tau )$. This is relevant in the scenario where both the wild-type and mutant populations have their growth restricted simultaneously by environmental factors, for example exposure to a chemotherapeutic agent. We observe that under a change of timescale this system is equivalent to our setting with exponential wild-type growth. This is due to the following argument.

In this setting, the wild-type population is governed by

18 $$\begin{aligned} \frac{\mathrm{d} n_{\tau }}{\mathrm{d} \tau }=\lambda z(\tau )n_{\tau }. \end{aligned}$$

Mutant clones are now initiated at a rate $\mu z(\tau ) n_{\tau }$. Let $\widehat{Z_{t}}$ be the size of a mutant population governed by the birth–death process with time-dependent rates. Once initiated, the size distribution obeys the forward Kolmogorov equation for time-dependent stochastic mutant proliferation

19 $$\begin{aligned} \begin{aligned} \partial _{t} {\mathbb {P}}(\widehat{Z_t}=k)&= z(t)(k-1){\mathbb {P}}(\widehat{Z_t}=k-1)\\&\quad + \beta z(t)(k+1) {\mathbb {P}}(\widehat{Z_t}=k+1)-(1+\beta ) z(t)k {\mathbb {P}}(\widehat{Z_t}=k). \end{aligned} \end{aligned}$$

If we let

$$\begin{aligned} F(\tau )=\int _{0}^{\tau }z(s)\hbox {d}s \end{aligned}$$

then under a new timescale, $\tau '=F^{-1}(\tau )$ , the mutant clone initiation will occur at a rate $\mu n_{\tau '}$. Further, using the chain rule to express (18) and (19) in terms of $\tau '$, we see that $n_{\tau '}=\hbox {e}^{\lambda \tau '}$ and that the forward Kolmogorov equation (19) becomes (1). Thus, under a time-rescaling, all dynamics are equivalent to the system with exponential wild-type growth and stochastic mutant proliferation with constant birth and death rates, as studied in this article or in Keller and Antal (2015).

### Poisson Process Characterisation of Tail

Complementing Corollary 3 in Sect. 5, following Tavare (1987), we can also describe the size distribution for large clones at long times via a Poisson process in the following way. Let $(Z^{(i)}(t))_{i\ge 1}$ be independent copies of the birth–death process as in Sect. 2 and $(T_{i})_{i\ge 1}\subset (0,\infty )$ be the points of a of Poisson process with intensity $\mu n_{\tau }$, for $\tau \ge 0$. The $T_{i}$ represent the clone arrival times, and so $K_t$ is the number of $T_{i}$ less than or equal to *t*.

Let us consider the size of the first clone. By known results about the large time behaviour of the birth–death process (Athreya and Ney 2004), as $t\rightarrow \infty$,

$$\begin{aligned} e^{-\lambda t}Z^{(1)}(t-T_{1})=e^{-\lambda T_{1}}e^{-\lambda (t-T_{1})}Z^{(1)}(t-T_{1})\rightarrow e^{-\lambda T_{1}}W_{1} \text{ a.s. } \end{aligned}$$

The distribution of the limiting random variable $W_{1}$ is composed of a point mass at 0 and an exponential random variable, precisely

$$\begin{aligned} {\mathbb {P}}(W_{1}\le x)=\beta +\lambda (1- e^{-\lambda x}),\quad x\ge 0. \end{aligned}$$

Analogously, with the details given in Tavare (1987) (Theorem 3), the limiting behaviour of the time-ordered clone sizes is given by

$$\begin{aligned} \lim _{t\rightarrow \infty } e^{-\lambda t}(Z^{(i)}(t-T_{i}))_{i\ge 1}=(e^{-\lambda T_{i}}W_{i})_{i\ge 1} \text{ a.s. } \end{aligned}$$

where $W_{1}$ is as before and all $W_{i}$ are *iid*. The random sequence $(e^{-\lambda T_{i}}W_{i})_{i\ge 1}$ takes non-negative real values; however, if we restrict our attention to only the positive elements (that is clones that do not die), then these can be taken to be points from a non-homogeneous Poisson process. More precisely, the set $\{\sigma _{j}\}_{j\ge 1}:=\{e^{-\lambda T_{i}}W_{i}\}_{i\ge 1}\setminus \{0\}$ are the points (in some order) from a Poisson process on $(0,\infty )$ with mean measure

20 $$\begin{aligned} m(x,\infty )=\mu \int _{x}^{\infty }n_{\lambda ^{-1}\log (s/x)}\frac{e^{-\lambda s}}{s}\,\hbox {d}s,\quad x>0. \end{aligned}$$

The proof of the above only requires minor modification from that of Theorem 4 in Tavare (1987).

The Poisson process description of the large clones, at large times, can also offer insight into further properties of the system, including links to the Poisson-Dirichlet distribution, see Tavare (1987), Durrett (2015). With regards to the present article, the interesting point is that for fixed $\varepsilon >0$, as the number of $\sigma _{j}>\varepsilon$ is finite almost surely, we may sample unformly from this set (i.e. $\{\sigma _{j}\}_{j\ge 1}\cap (\varepsilon ,\infty )$) and construct a random variable $Y_{\varepsilon }$ with distribution

$$\begin{aligned} {\mathbb {P}}(Y_{\varepsilon }>x)=\frac{m(x,\infty )}{m(\varepsilon ,\infty )},\quad x\ge \varepsilon \end{aligned}$$

where $m(x,\infty )$ is as in (20). The new variable $Y_{\varepsilon }$ can be related to the previously considered random variable $Y_t$ by the following result, whose proof is contained in “Appendix F”.

#### Theorem 6

For $\varepsilon >0$, with $Y_{\varepsilon }$ as above,

$$\begin{aligned} \lim _{t\rightarrow \infty }{\mathbb {P}}(Y_{t}e^{-\lambda t}>x|Y_{t}e^{-\lambda t}>\varepsilon )={\mathbb {P}}(Y_{\varepsilon }>x),\quad x\ge \varepsilon . \end{aligned}$$

Of note is the reappearance of power-law behaviour with a cut-off in the density of $Y_{\varepsilon }$. For example in the constant wild-type case, $n_{\tau }=1$, the density, using (20), is given by

$$\begin{aligned} f_{Y_{\varepsilon }}(x)=\frac{d}{dx}{\mathbb {P}}(Y_{\varepsilon }\le x)=\frac{e^{-\lambda x}}{x\varGamma (0,\lambda \varepsilon )},\quad x\ge \varepsilon . \end{aligned}$$

For exponential growth with neutral mutants, $n_{\tau }=e^{\lambda \tau }$,

$$\begin{aligned} f_{Y_{\varepsilon }}(x)=\frac{e^{-\lambda x}}{x^2 }(1+\lambda x)\varepsilon e^{\lambda \varepsilon },\quad x\ge \varepsilon . \end{aligned}$$

Note that the exponents in the power-law terms is equal to that given in Corollary 3, indicating the two approaches are complimentary.

## Discussion

In this study, we focus on the size distribution for mutant clones initiated at a rate proportional to the size of the wild-type population. The size of the wild-type population is dictated by a generic deterministic growth function, and the mutant growth is stochastic. This shifts the focus from previous studies which have mostly been concerned with exponential, or mean exponential, wild-type growth, and considered the total number of mutants. Results for the total number of mutants are, however, easily obtained from the clone size distribution.

The special cases of exponential, power-law and logistic wild-type growth were treated in detail, due to their extensive use in models for various applications. Utilising a generating function centred approach, exact time-dependent formulas were ascertained for the probability distributions in each case. Regardless of the growth function, the mass function is monotone decreasing and the distribution has a cut-off for any finite time. This cut-off goes to infinity for large times and is often enormous in practical applications; hence, we focused on the approach to the cut-off.

We found that the clone size distribution behaves quite distinctly for exponential-type versus subexponential wild-type growth. Although the probability of finding a clone of any given size stays finite as $t\rightarrow \infty$ for exponential-type growth, it tends to zero for subexponential type. Despite these differences, with a proper scaling, for a large class of growth functions, we proved that the clone size distribution has a universal long-time form. This long-time form possesses a power-law “fat” tail which decays as 1 / *k* for subexponential wild-type growth, but faster for exponential-type growth. This can be intuitively understood as the tail distribution represents clones that arrive early, and the chance that a clone is initiated early in the process is larger for a slower growing wild-type function. Hence, we expect a “fatter” tail in the subexponential case.

Note that although we consider the case of subexponential wild-type growth, surviving mutant clones will grow exponentially for large time, which can be unrealistic in some situations. Stochastic growth which accounts for environmental restrictions, for instance the logistic branching process, introduces further technical difficulties and is left for future work. We do note that, despite the drawbacks of deterministic mutant growth as discussed in Sect. 7, when both the wild-type and mutant populations grow deterministically as $\tau ^{\rho }$, it is easy to see that for large times the clone size distribution still displays a power-law tail, $\lim _{t\rightarrow \infty } t f_{Y_t}(y) = \frac{\rho +1}{\rho }y^{1/\rho -1}.$

An underlying motivation for this work is the scenario of primary tumours spawning metastases in cancer. We test our hypothesis regarding a power-law tail in metastasis size distributions by analysing empirical data. For 19 of 21 data-sets, the power-law distribution is deemed more likely than an exponentially decaying distribution. The exponent of the power-law decay was estimated in each case and found to lie between $-1$ and $-2$. Interpreting this in light of our theory, either the primary tumour had entered a subexponential growth phase or, if one assumes exponential primary growth, the metastatic cells had a fitness advantage compared to those in the primary. Either way we can conclude that, for the majority of patients, the metastases grew faster than the primary tumour.

## Appendix A: Special Functions, Definitions and Requisite Results

Required definitions and identities taken from DLMF (2016) unless otherwise stated.

With $s,z\in {\mathbb {C}}$ the polylogarithm of order *s* is defined as

$$\begin{aligned} {\mathrm {Li}}_{s}(z)=\sum _{k\ge 1}\frac{z^{k}}{k^{s}}. \end{aligned}$$

Note that ${\mathrm {Li}}_{1}(z)=-\log (1-z)$. A required identity (from Weisstein 2016) is

21 $$\begin{aligned} {\mathrm {Li}}_{-n}(z)=\sum _{k=0}^{n}k! S(n+1,k+1)\bigg (\frac{z}{1-z}\bigg )^{k+1} \end{aligned}$$

for $n\in {\mathbb {N}}$. Here, *S*(*n*, *k*) are the Stirling numbers of the second kind.

Gauss’s hypergeometric function also appears and for complex *a*, *b*, *c*, *z* is defined by the power series

$$\begin{aligned} \mathop {F}\nolimits \!\left( \genfrac{}{}{0.0pt}0{a,b}{c};z\right) =\sum _{k\ge 0}\frac{(a)_{k}(b)_{k}}{(c)_{k}}\frac{z^{k}}{k!}\quad \text {for} \, |z|<1, \end{aligned}$$

and by analytic continuation elsewhere. Here, $(a)_{k}$ denotes the Pochhammer symbol or rising factorial, that is

$$\begin{aligned} (a)_{k}=\frac{\varGamma (a+k)}{\varGamma (a)}=a(a+1)(a+2)\cdots (a+k-1). \end{aligned}$$

Some required identities for the hypergeometric function are:

22 $$\begin{aligned} \mathop {F}\nolimits \!\left( \genfrac{}{}{0.0pt}0{a,b}{c};z\right)= & {} (1-z)^{-b}\mathop {F}\nolimits \!\left( \genfrac{}{}{0.0pt}0{c-a,b}{c};\frac{z}{z-1}\right) , \end{aligned}$$

23 $$\begin{aligned} \mathop {F}\nolimits \!\left( \genfrac{}{}{0.0pt}0{1,b}{c};z\right)= & {} 1+\frac{b}{c}z\mathop {F}\nolimits \!\left( \genfrac{}{}{0.0pt}0{1,b+1}{c+1};z\right) , \end{aligned}$$

24 $$\begin{aligned} \mathop {F}\nolimits \!\left( \genfrac{}{}{0.0pt}0{1,1}{2};z\right)= & {} -\frac{\log (1-z)}{z}, \end{aligned}$$

and the following connection can be made to the incomplete beta-function

25 $$\begin{aligned} \frac{z^{a}}{a}\mathop {F}\nolimits \!\left( \genfrac{}{}{0.0pt}0{a,1-b}{a+1};z\right) =B_{z}(a,b)=\int _{0}^{x}t^{a-1}(1-t)^{b-1}\,\hbox {d}t. \end{aligned}$$

For any analytic function $f(z)=\sum _{n\ge 0}f_{n}z^{n}$, we denote the n*t*h coefficient as $[z^{n}]f(z)=f_{n}.$

### Theorem A1

(Flajolet and Sedgewick 2009: Exponential Growth Formula) If *f*(*z*) is analytic at 0 and *R* is the modulus of a singularity nearest the origin in the sense that $R:=\sup \{r\ge 0 |\text { f is analytic in }|z|<r\}.$ Then the coefficient $[z^{n}]f(z)$ satisfies $f_{n}=R^{-n}\varTheta (n)$ where $\limsup _{n}\root n \of {|\varTheta (n)|}=1$.

We utilise several results from Bingham et al. (1987) on the theory of regularly varying functions which we now define.

### Definition 2

(Bingham et al. 1987) A Lebesgue measurable function $f:{\mathbb {R}}^+\mapsto {\mathbb {R}}$ that is eventually positive is *regularly varying* (at infinity) if for some $\kappa \in {\mathbb {R}}$,

$$\begin{aligned} \lim _{t\rightarrow \infty } \frac{f(tx)}{f(t)}=x^{\kappa }, \quad x>0. \end{aligned}$$

The notation $f\in RV_{\kappa }$ will be used and we will denote $f\in RV_{0}$ as *slowly varying functions*.

### Theorem A2

(Bingham et al. 1987: Characterisation Theorem) Suppose $f:{\mathbb {R}}^{+}\mapsto {\mathbb {R}}$ is measurable, eventually positive, and $\lim _{t\rightarrow \infty } \frac{f(tx)}{f(t)}$ exists, and is finite and positive for all *x* in a set of positive Lebesgue measure. Then, for some $\kappa \in {\mathbb {R}}$,

(i) $$ f\in RV_{\kappa }$$.
(ii) $$f(y)=y^{\kappa }l(y)$$ where $$l\in RV_{0}$$.

### Proposition A1

(Bingham et al. 1987: Proposition 1.3.6) For $f\in RV_{\kappa }$ $\lim _{t\rightarrow \infty }\frac{ \log f(t)}{\log t }= \kappa .$

### Theorem A3

(Bingham et al. 1987: Karamata’s Theorem) If $f\in RV_{\kappa }$, *X* sufficiently large such that *f*(*y*) is locally bounded in $[X,\infty )$, and $\kappa >-1$, then

$$\begin{aligned} \int _{X}^{y}f(s)\,\hbox {d}s\sim \frac{yf(y)}{\kappa +1}\quad \text {as} \quad y\rightarrow \infty . \end{aligned}$$

### Proposition A2

(Bingham et al. 1987: Proposition 1.5.9.a) Let $l\in RV_{0}$ and choose *X* so that *l* is locally integrable on $[X,\infty )$ . Then,

(i) $$\int _{X}^{x}\frac{l(t)}{t}\,\hbox {d}t \in RV_{0} $$ .
(ii) $$\frac{1}{l(x)}\int _{X}^{x}\frac{l(t)}{t}\,\hbox {d}t\rightarrow \infty \text { as } x\rightarrow \infty $$.

## Appendix B: Proofs for Section 3

In this work, we have fixed the birth rate to be one. Other works, for example Keller and Antal (2015), use a birth–death process with birth rate $\alpha '$ and death rate $\beta '$ under timescale $t'$. Then, the timescale used in the present work is defined by $t=\alpha ' t'$. This in turn implies that all rates under *t* are given by dividing the corresponding rate under $t'$ by $\alpha '$, e.g. $\beta =\frac{\beta '}{\alpha '}$.

### Lemma B1

Consider generating functions $F(s)=\sum _{n\ge 0}p_n s^n$ and $G(s)=\sum _{n\ge 0}q_n s^n$ where $F(s)=e^{G(s)}$. Then $p_0=e^{q_0}$ and for $n\ge 1$ the following recursion holds

$$\begin{aligned} np_n = \sum _{k=0}^{n-1} (n-k) p_k q_{n-k} \ . \end{aligned}$$

### Proof

Clearly, $p_0=e^{q_0}$ from $F(0)=e^{G(0)}$. By differentiating *F*(*s*), we obtain $F'(s)= F(s) G'(s)$, and in general

$$\begin{aligned} F^{(n)}(s) = \sum _{k=0}^{n-1} \left( {\begin{array}{c}n-1\\ k\end{array}}\right) F^{(k)}(s) G^{(n-k)}(s) \end{aligned}$$

which can be shown by induction using Pascal’s formula for binomial coefficients. Evaluating the above equation at $s=0$ and using that $F^{(m)}(0)=m! p_n$ and $G^{(m)}(0)=m! q_n$ we arrive at the announced recursion. $\square$

### Proof of Proposition 1

Utilising generating functions,

$$\begin{aligned} \begin{aligned} {\mathbb {E}}(s^{B_{t}}|B_{t}>0)&=\frac{\mathcal {B}_t(s)-\mathcal {B}_t(0)}{1-\mathcal {B}_t(0)} =\frac{e^{{\mathbb {E}}(K_{t}) (\mathcal {Y}_t(s)-1)}-e^{{\mathbb {E}}(K_{t}) (\mathcal {Y}_t(0)-1)}}{1-e^{{\mathbb {E}}(K_{t}) (\mathcal {Y}_t(0)-1)}} \\&=\frac{{\mathbb {E}}(K_{t}) (\mathcal {Y}_t(s)-1)-(\mathcal {Y}_t(0)-1))}{-{\mathbb {E}}(K_{t}) (\mathcal {Y}_t(0)-1)}+O({\mathbb {E}}(K_{t}) ) \\&=\frac{\mathcal {Y}_t(s)-\mathcal {Y}_t(0)}{1-\mathcal {Y}_t(0)}+O({\mathbb {E}}(K_{t}) )={\mathbb {E}}(s^{Y_{t}}|Y_{t}>0)+O({\mathbb {E}}(K_{t}) ). \end{aligned} \end{aligned}$$

$$\square $$

## Appendix C: Proofs for Section 4

We derive the generating function for the clone size distribution for stochastic growth and power-law wild-type growth, $n_{\tau }=\tau ^{\rho }$, given in (13). From (9), we have

$$\begin{aligned} \mathcal {Y}_t(s)= & {} \frac{\rho +1}{t^{\rho +1}}\int _{0}^{t}\tau ^{\rho }\bigg (1-\frac{\lambda }{1-\xi e^{-\lambda (t-\tau )}}\bigg )\, \hbox {d}\tau \\= & {} 1-\frac{(\rho +1)\lambda }{t^{\rho +1}}\int _{0}^{t}\frac{\tau ^{\rho }}{1-\xi e^{-\lambda (t-\tau )}}\,\hbox {d}\tau . \end{aligned}$$

It is enough to show

26 $$\begin{aligned} \int \frac{\tau ^{\rho }}{1-\xi e^{-\lambda (t-\tau )}}\,\hbox {d}\tau = \frac{\tau ^{\rho +1}}{\rho +1}+\rho ! \sum _{i=0}^{\rho }\frac{(-1)^{i}}{(\rho -i)!\lambda ^{i+1}}\tau ^{\rho -i}{\mathrm {Li}}_{i+1}(\xi e^{-\lambda (t-\tau )})+C\nonumber \\ \end{aligned}$$

where *C* is a constant of integration. This may be derived by a binomial expansion of the denominator and an identity for the incomplete gamma function, but for succinctness we simply differentiate both sides with respect to $\tau$. First, we note that

$$\begin{aligned} z \partial _{z} {\mathrm {Li}}_{i}(z)={\mathrm {Li}}_{i-1}(z). \end{aligned}$$

Now differentiating the right hand side of (26) yields

$$\begin{aligned}&\tau ^{\rho } +\frac{\tau ^{\rho }\lambda {\mathrm {Li}}_{0}(\xi e^{-\lambda (t-\tau )})}{\lambda }+ \rho ! \sum _{j=0}^{\rho -1} \frac{(-1)^{j}(\rho -j)\tau ^{\rho -j-1}{\mathrm {Li}}_{j+1}(\xi e^{-\lambda (t-\tau )})}{(\rho -j)! \lambda ^{j+1}} \nonumber \\&\qquad +\,\rho !\sum _{i=1}^{\rho }\frac{(-1)^{i} \tau ^{\rho -i}\lambda {\mathrm {Li}}_{i}(\xi e^{-\lambda (t-\tau )})}{(\rho -i)!\lambda ^{i+1}}=\tau ^{\rho }(1+{\mathrm {Li}}_{0}(\xi e^{-\lambda (t-\tau )})) \end{aligned}$$

where the equality follows by the telescoping nature of the sums. Noting that $(1-\xi e^{-\lambda (t-\tau )})^{-1}={\mathrm {Li}}_{0}\left( \xi e^{-\lambda (t-\tau )}\right) +1$ and applying the limits of the integral gives the desired result.

To determine the mass function, we seek a power series representation of the generating function. We focus on the $\beta =0$ case and thus $\xi =\frac{s}{s-1}$. By the definition of the polylogarithm and the binomial theorem

$$\begin{aligned} {\mathrm {Li}}_{i}\left( \frac{s}{s-1}\right) =\sum _{k\ge 1}\sum _{j\ge 0}{k+j-1\atopwithdelims ()j}(-1)^{k}\frac{s^{j+k}}{k^i}. \end{aligned}$$

Reindexing the sum, we obtain

$$\begin{aligned} {\mathrm {Li}}_{i}\left( \frac{s}{s-1}\right) =\sum _{m\ge 1}s^{m}\sum _{k=1}^{m}{m-1\atopwithdelims ()m-k}\frac{(-1)^k}{k^i}\quad \text {and}\quad {\mathrm {Li}}_{i}\left( \frac{se^{-t}}{s-1}\right) =\sum _{m\ge 1}s^{m}\sum _{k=1}^{m}{m-1\atopwithdelims ()m-k}\frac{(-e^{-t})^k}{k^i}. \end{aligned}$$

Applying this to the polylogarithmic terms in $\mathcal {Y}_t(s)$, and noting

$$\begin{aligned} \sum _{k=1}^{m}{m-1\atopwithdelims ()m-k}\frac{(-1)^k}{k^i}=\frac{1}{m}\sum _{k=1}^{m}{m\atopwithdelims ()k}\frac{(-1)^k}{k^{i-1}}\quad \text {and}\quad \sum _{k=1}^{m}{m\atopwithdelims ()k}(-1)^k=-1, \end{aligned}$$

yields (14) as the desired mass function.

### Proof of Proposition 2

Using (8), we see that for $k\ge 1$

$$\begin{aligned} {\mathbb {P}}(Y_{t}=k+1)-{\mathbb {P}}(Y_{t}=k)=\frac{1}{a_t}\int _{0}^{t}n_{t-\tau }\left[ {\mathbb {P}}(Z_{\tau }=k+1)-{\mathbb {P}}(Z_{\tau }=k)\right] \,\hbox {d}\tau . \end{aligned}$$

Now from (3), it is clear that the integrand is negative for finite, positive $\tau$ giving the result. $\square$

### Proof of Theorem 1

The result is an application of Theorem A1. We seek the closest to the origin singularity of

$$\begin{aligned} I_{t}(s)=\int _{0}^{t}n_{\tau }{\mathcal {Z}}_{t-\tau }(s)\,\hbox {d}\tau =\int _{0}^{t}n_{t-\tau }{\mathcal {Z}}_{\tau }(s)\,\hbox {d}\tau \end{aligned}$$

which is claimed to be at ${\mathcal {S}}_{t}$. Indeed, we note that for $|s|<{\mathcal {S}}_{t}$, ${\mathcal {Z}}_{\tau }(s)$ is analytic for all $\tau$, and as $n_{\tau }$ is continuous, we can conclude that the $I_{t}(s)$ is analytic in this region also [Chapter 2, Theorem 5.4 in Stein and Shakarchi (2003)]. As $n_{\tau }>0$ there exists $\varepsilon >0$ such that

$$\begin{aligned} |I_{t}(s)|\ge \varepsilon \bigg |\int _{0}^{t}{\mathcal {Z}}_{\tau }(s)\,\hbox {d}\tau \bigg |=\varepsilon \bigg |\beta t+\log \bigg [\frac{\lambda }{1-\beta e^{-\lambda t}-s(1-e^{-\lambda t})}\bigg ]\bigg |. \end{aligned}$$

The rightmost expression can be seen to have closest to origin singularity at ${\mathcal {S}}_{t}$ and as $a_{t}\mathcal {Y}_t(s)=I_{t}(s)$, by Theorem A1, we can conclude Theorem 1. $\square$

## Appendix D: Proofs for Section 5

### Proof of Lemma 1

Choose $x\ge 0$ and let $y=e^{t},\,c=e^{-x}$. Consider the function $g(z)=n_{\log (z)}$. Then Theorem A2(i) yields

$$\begin{aligned} \lim _{t\rightarrow \infty }\frac{n_{t-x}}{n_{t}}=\lim _{y\rightarrow \infty }\frac{g(yc)}{g(y)}=c^{\delta ^*}=e^{-x\delta ^*}. \end{aligned}$$

Further, Proposition A1 gives

$$\begin{aligned} \lim _{y\rightarrow \infty }\frac{\log g(y)}{\log y }=\lim _{t\rightarrow \infty }\frac{\log (n_{t})}{t}= \delta ^*\ge 0. \end{aligned}$$

The non-negativity of $\delta ^*$ is dictated by the monotone increasing nature of $n_{\tau }$. $\square$

To prove Theorem 2, we require the following:

### Lemma D1

Let $s_{1}(t),\,s_{2}(t)$ be subexponential functions, then

(i) $$n_{t}=e^{t\delta ^* }s_{1}(t)$$.
(ii) For $$\eta \ge 0,\,C>0$$
  $$\begin{aligned} \int _{0}^{t}n_{\tau }e^{-\eta \tau }\,\hbox {d}\tau \sim {\left\{ \begin{array}{ll} \frac{e^{(\delta ^*-\eta ) t}s_{1}(t)}{\delta ^*-\eta } &{}\quad \eta<\delta ^* \\ s_{2}(t) &{}\quad \delta ^*=\eta \\ C &{}\quad \delta ^*<\eta \end{array}\right. } \quad \text {as } t\rightarrow \infty . \end{aligned}$$

We highlight that neither subexponential function depend on $\eta$.

### Proof

(i) For $y=e^{t}$, $n_{\log y}=g(y)$. Now $g\in RV_{\delta ^*}$ hence $g(y)=y^{\delta ^*}l(y)$ with $l\in RV_{0}$ by Theorem A2(ii). Setting $s_1(\log y)=l(y)$, by Lemma 1, $s_{1}(t)$ is subexponential. (ii) Let *g*(*y*) be as above. With $\delta ^* > \eta \ge 0$ and using the change of variables $s=\log \tau$ we have

27 $$\begin{aligned} \int _{0}^{t}n_{\tau }e^{-\eta \tau }\,\hbox {d}\tau =\int _{1}^{y}g(s)s^{-1-\eta }\,\hbox {d}s \sim \frac{y^{-\eta }g(y)}{\delta ^*-\eta }=\frac{e^{(\delta ^*-\eta )t}s_1(t)}{\delta ^*-\eta } \end{aligned}$$

where the asymptotic equivalence is due to Theorem A3 applied to $g(y)y^{-1-\eta }\in RV_{\delta ^*-\eta -1}$ and the final equality is by part (i). For $\delta ^*=\eta$, by Theorem A2(ii) the integrand will be a subexponential function. Applying the same change of variables as in (27), we see by Proposition A2(i) that the integral is a slowly varying function in *y* and hence is subexponential in *t*, which we denote $s_{2}(t)$. Now for $\delta ^*<\eta$, by Lemma 1, we may choose *t* large enough such that $n_{t}^{1/t}<e^{(\delta ^*+\eta )/2}$ which by a basic result for Laplace transforms, see, e.g. Theorem 1.11 in Schiff (1999), ensures convergence to a finite, positive constant. $\square$

As an example, which will be useful for the next proof, we apply the above lemma to $a_{t}$. With $s_{1}(t),\,s_{2}(t)$ subexponential functions

$$\begin{aligned} a_{t}=\int _{0}^{t}n_{\tau }\,\hbox {d}\tau \sim {\left\{ \begin{array}{ll} \frac{e^{\delta ^* t}s_{1}(t)}{\delta ^*} &{}\quad \delta ^* >0 \\ s_{2}(t) &{}\quad \delta ^*=0 \end{array}\right. } \quad \text {as }t\rightarrow \infty . \end{aligned}$$

### Proof of Theorem 2

We require the first and second moments of $Z_{t}$ which may be found by differentiating (2), or see Lemma F1. Then

28 $$\begin{aligned} {\mathbb {E}}(Y_{t})= & {} \frac{1}{a_{t}}\int _{0}^{t}n_{\tau }{\mathbb {E}}(Z_{t-\tau })\,\hbox {d}\tau =e^{\lambda t}\frac{\int _{0}^{t}n_{\tau }e^{-\lambda \tau }\,\hbox {d}\tau }{\int _{0}^{t}n_{\tau }\,\hbox {d}\tau }, \end{aligned}$$

29 $$\begin{aligned} {\mathbb {E}}(Y_{t}^2)= & {} \frac{1}{a_{t}}\int _{0}^{t}n_{\tau }{\mathbb {E}}(Z_{t-\tau }^2)\,\hbox {d}\tau \nonumber \\= & {} \frac{e^{2\lambda t}}{a_{t}\lambda }\left( 2\int _{0}^{t}n_{\tau }e^{-2\lambda \tau }\,\hbox {d}\tau -(2-\lambda )e^{-\lambda t}\int _{0}^{t}n_{\tau }e^{-\lambda \tau }\,\hbox {d}\tau \right) . \end{aligned}$$

Throughout let $s_{t}$ be a generic subexponential function and it will be helpful to observe that the reciprocal or constant multiples of a subexponential function are subexponential. We first consider the mean. For the cases $\delta ^*\ne \lambda$, applying Lemma D1(ii) to (28) with $\eta =\lambda$ for the numerator and $\eta =0$ for the denominator proves the claim. For $\delta ^*=\lambda$, using Lemma D1(i) then (ii), we have

30 $$\begin{aligned} {\mathbb {E}}(Y_{t})=\frac{e^{\delta ^* t}\int _{0}^{t}e^{\delta ^* \tau }s_{\tau }e^{-\delta ^*\tau }}{\int _{0}^{t}e^{\delta ^*\tau }s_{\tau }\,\hbox {d}\tau }\sim \frac{\delta ^*\int _{0}^{t}s_{\tau }\,\hbox {d}\tau }{s_{t}}=s_{1}(t). \end{aligned}$$

That $s_{1}(t)$ diverges can be seen by applying the standard change of variables $t=\log (y),\,\tau =\log (s)$ coupled with Proposition A2(ii). Turning to the variance, with ${\text {Var}}(Y_{t})={\mathbb {E}}(Y_{t}^2)-{\mathbb {E}}(Y_{t})^2$, when $\delta ^*>2\lambda$ we apply Lemma D1(ii) term by term to (29). For $\delta ^*<\lambda$ all integrals converge and so, with $C_{1},\,C_{2}$ constants,

$$\begin{aligned} {\mathrm {Var}}(Y_t)\sim \frac{e^{2\lambda t}}{a_{t}}\left( C_{1}-\frac{C_{2}}{a_{t}}\right) \sim C_{1}\frac{e^{2\lambda t}}{a_{t}}. \end{aligned}$$

The last relation is due to the monotonicity of $a_{t}$ and the desired representation is obtained by applying Lemma D1(ii) to $a_{t}$ and absorbing $C_{1}$ into $s_{4}(t)$. When $\lambda \le \delta ^*<2\lambda$, the same argument holds as long as we note that

$$\begin{aligned} e^{-\lambda t}\int _{0}^{t}n_{\tau }e^{-\lambda t}\,\hbox {d}\tau =e^{-\lambda t}\int _{0}^{t}e^{(\delta ^*-\lambda )\tau }s_{\tau }\,\hbox {d}\tau \le e^{-(2\lambda -\delta ^*)t}\int _{0}^{t}s_{\tau }\,\hbox {d}\tau . \end{aligned}$$

By Proposition A2(i) the rightmost integral is a subexponential function and as we may always choose *t* sufficiently large such that $s_{t}^{1/t}<e^{2\lambda -\delta ^*}$, we find

$$\begin{aligned} e^{-\lambda t}\int _{0}^{t}n_{\tau }e^{-\lambda t}\,\hbox {d}\tau \rightarrow 0. \end{aligned}$$

Applying Lemma D1 to $a_{t}^{-1}\int _{0}^{t}n_{\tau }e^{-\lambda t}\,\hbox {d}\tau$ demonstrates the contribution from the mean squared is negligible. For $\delta ^*=2\lambda$, we apply the same argument as in (30) to each term and this completes the proof. $\square$

In order to prove Theorem 3, we require the following lemma.

### Lemma D2

For $|s|<1,\,\beta \in [0,1)$, $u\in [0,1]$ and $\xi$ as in (2), we have

$$\begin{aligned} \left| \frac{\xi }{1-\xi u}\right| \le \left| \frac{\beta -s}{1-\max \{\beta ,|s|\}}\right| . \end{aligned}$$

### Proof

By the definition of $\xi$,

$$\begin{aligned} \frac{\xi }{1-\xi u}=\frac{\beta -s}{1-s-(\beta -s)u}. \end{aligned}$$

The triangle inequality yields

$$\begin{aligned} |1-s-\beta u+su|=|1-(\beta u+s(1-u))|\ge |1-|\beta u+s(1-u)|| \end{aligned}$$

and

$$\begin{aligned} |\beta u+s(1-u)|\le u\beta +(1-u)|s|\le \max \{\beta ,|s|\}. \end{aligned}$$

The claimed inequality now follows. $\square$

### Proof of Theorem 3

To avoid division by 0 let $t>0$. Taking the generating function for $Y_{t}$ from equation (9), we apply the change of variables $u=e^{-\lambda \tau }$ which gives

$$\begin{aligned} \mathcal {Y}_t(s)-\beta =-\frac{1}{a_{t}}\int _{e^{-\lambda t}}^{1}n_{t+\frac{\log u}{\lambda }}\frac{\xi }{1-\xi u}\,\hbox {d}u. \end{aligned}$$

Now recalling $n_{\tau }=0$ for $\tau <0$ and multiplying both sides by $\frac{a_t}{n_t}$ yields

$$\begin{aligned} \frac{a_{t}}{n_t}(\mathcal {Y}_t(s)-\beta )=-\int _{0}^{1}\frac{n_{t+\frac{\log u}{\lambda }}}{n_{t}}\frac{\xi }{1-\xi u}\,\hbox {d}u. \end{aligned}$$

Noting that by monotonicity $n_{t+\frac{\log u}{\lambda }}\big /n_{t}\le 1,$ which coupled with Lemma D2 shows the integrand may be dominated. By assumption, the integrand converges, and therefore, using Lemma 1 and the dominated convergence theorem, we have

$$\begin{aligned} \lim _{t\rightarrow \infty }\frac{a_{t}}{n_t}(\mathcal {Y}_t(s)-\beta )= & {} -\int _{0}^{1}u^{\delta ^*/\lambda }\frac{\xi }{1-\xi u}\,\hbox {d}u =\frac{-1}{\xi ^{\gamma ^*}}\mathrm {B}_{\xi }(\gamma ^*+1,0) \nonumber \\= & {} \frac{1}{\gamma ^*}\left[ 1-\mathop {F}\nolimits \!\left( \genfrac{}{}{0.0pt}0{1,\gamma ^*}{1+\gamma ^*};\xi \right) \right] . \end{aligned}$$

The final equality follows from applying (25) then (23). $\square$

### Proof of Corollary 1

The first statement is given by applying Lemma D1(ii) to $a_t/n_t$. Then, taking the limiting generating function in Theorem 3, we firstly apply (23) then (24) which yields generating function representation for $\gamma ^*=0$. The mass function for $\gamma ^*=0$ is simply a logarithmic expansion. For $\gamma ^*>0$, we use the expression given in Appendix A of Keller and Antal (2015) to obtain a series expansion for the generating function in terms of *s*, and the coefficients of the expansion give the mass function. $\square$

### Proof of Corollary 3

The analysis involves expanding the limiting generating function in Theorem 3 around its singularity at $s=1$ and exactly mirrors that given in section 6 of Keller and Antal (2015) and so is omitted. $\square$

### Proof of Theorem 4

The mean and variance can be obtained by using (11) with Theorem 2 (the second moment dominates the mean squared in all divergent cases). For the generating function, (10) gives

$$\begin{aligned} \mathcal {B}_t(s)=\exp \left[ \mu a_{t}(\mathcal {Y}_t(s)-1)\right] =\exp \left[ \theta \frac{a_{t}}{n_{t}}(\mathcal {Y}_t(s)-\beta +\beta -1)\right] \end{aligned}$$

which coupled with Theorem 3 yields the result. The map in (10) is analytic so the tail can be obtained by its expansion coupled with Corollary 3. $\square$

## Appendix E: Maximum Likelihood Estimators for Distributions Considered

Consider a data-set $\mathbf y =(y_{1},\ldots ,y_{N})$ assumed to be a realisation of the random vector $\mathbf Y _{t}=(Y^{(1)}_{t},\ldots ,Y^{(N)}_{t})$ where all $Y_{t}^{(i)}$ are $\textit{iid}$ random variables representing the metastasis sizes and let $m=\text {min}(\mathbf y )$. Then the maximum likelihood estimator (MLE) $\hat{\omega }$ for a one parameter probability distribution ${\mathbb {P}}(\mathbf Y _{t}=\mathbf y ;\omega )$ is

$$\begin{aligned} \hat{\omega }= & {} {\mathop {{{\mathrm{arg\,max}}}}\limits _{{\omega }}} \log (\mathfrak {L}(\omega ))= {\mathop {{{\mathrm{arg\,max}}}}\limits _{{\omega }}} \log ({\mathbb {P}}(\mathbf Y _{t}=\mathbf y ;\omega ))\\= & {} {\mathop {{{\mathrm{arg\,max}}}}\limits _{\omega }} \log \bigg [\prod _{i=1}^{N}{\mathbb {P}}(Y_{t}^{(i)}=y_{i};\omega )\bigg ] \end{aligned}$$

where $\mathfrak {L}(\omega )$ is the likelihood function and the joint distribution becomes a product by independence. We derive the MLE under the assumption that the data is sampled from a distribution whose tail follows the geometric distribution, the power-law case is analogous but for the final step.

Assume for at least $y\ge m$, that ${\mathbb {P}}_{2}(Y_t=y;p)=C_{2}p(1-p)^{y}$. Let $A={\mathbb {P}}(Y_{t}<m)$ (no indices are required as this quantity is assumed independent of the tail) then

$$\begin{aligned} \sum _{y\ge m} {\mathbb {P}}_{2}(Y_t=y;p)=\sum _{y\ge m}C_{2}p(1-p)^y=1-A \implies C_{2}=(1-A)(1-p)^{-m}. \end{aligned}$$

The log-likelihood is now given by

$$\begin{aligned} \log \mathfrak {L}(p)=N\log (1-A)-mN\log (1-p)+N\log (p)+\log (1-p)\sum _{i=1}^{N}y_{i}. \end{aligned}$$

Setting $\partial _{p}\log \mathfrak {L}(p)=0$, we solve to find the MLE

$$\begin{aligned} \hat{p}=\frac{N}{\sum _{i=1}^{N}y_{i}+N(1-m)}. \end{aligned}$$

The power-law case is analogous. There $C_{1}=\frac{1-A}{\zeta (\omega ,m)}$, where $\zeta$ is the Hurwitz zeta function. No closed form expression is found for the MLE and instead we have the approximation (Clauset et al. 2009)

$$\begin{aligned} \hat{\omega }\approx 1+N\bigg [\sum _{i=1}^{N}\log \bigg (\frac{y_{i}}{m-\frac{1}{2}}\bigg )\bigg ]^{-1}. \end{aligned}$$

This was used for the estimates given in Sect. 6.

## Appendix F: Proofs for Section 7

In order to prove Theorem 5, we require the following lemma regarding the moments of the birth–death process:

### Lemma F1

$$\begin{aligned} {\mathbb {E}}(Z_{t}^{m})=\frac{\lambda }{(1-\beta e^{-\lambda t})}\sum _{k=0}^{m}k!S(m+1,k+1)\bigg (\frac{e^{\lambda t}-1}{\lambda }\bigg )^{k}, \end{aligned}$$

where *S*(*m*, *k*) are Stirling numbers of the second kind. Hence, as $t\rightarrow \infty$,

$$\begin{aligned} {\mathbb {E}}(Z_{t}^m)=m!e^{m\lambda t}\lambda ^{-(m-1)}+O(e^{(m-1)\lambda t}). \end{aligned}$$

### Proof of Lemma F1

Recall the generating function for the birth–death process (2) whose power series representation has coefficients given by (3). The moment generating function is $M_{Z_{t}}(s)={\mathcal {Z}}_{t}(e^{s})$ and hence

$$\begin{aligned} {\mathcal {Z}}_{t}(e^{s})-{\mathcal {Z}}_{t}(0)=\left( 1-\beta {\mathcal {S}}_{t}^{-1}\right) ({\mathcal {S}}_{t}-1)\sum _{j\ge 1}{\mathcal {S}}_{t}^{-j}e^{sj}. \end{aligned}$$

Thus for $$m\ge 1$$

$$\begin{aligned} {\mathbb {E}}(Z_{t}^{m})=\partial _{s}^{m}{\mathcal {Z}}_{t}(e^{s})|_{s=0}=\left( 1-\beta {\mathcal {S}}_{t}^{-1}\right) ({\mathcal {S}}_{t}-1)\sum _{j\ge 1}{\mathcal {S}}_{t}^{-j}j^{m}. \end{aligned}$$

Since $\sum _{j\ge 1}{\mathcal {S}}_{t}^{-j}j^{m}={\mathrm {Li}}_{-m}({\mathcal {S}}_{t}^{-1})$, we can use (21) and thus arrive at our first result. Note $S(m,m)=1$ and so focusing on the leading order in *t*, the summand with $k=m$ is $m!\bigg (\frac{e^{\lambda t}-1}{\lambda }\bigg )^{m}=m!e^{m\lambda t}\lambda ^{-m}+O(e^{(m-1)\lambda t})$, which proves the claim. $\square$

### Proof of Theorem 5

In the deterministic case, we have

$$\begin{aligned} {\mathbb {E}}((Y_{t}^{\mathrm {Det}})^{m})=\frac{1}{a_{t}}\int _{0}^{t}n_{\tau }e^{m\lambda (t-\tau )}\,\hbox {d}\tau . \end{aligned}$$

The moments for stochastic mutant growth are obtained from the moment generating function $M_{Y_{t}}(s)=\mathcal {Y}_t(e^{s})$. The moments are therefore

$$\begin{aligned} {\mathbb {E}}((Y_{t}^{\mathrm {Stoch}})^{m})=\partial _{s}^{m}M_{Y_{t}}(0)=\frac{1}{a_{t}}\int _{0}^{t}n_{\tau }\partial _{s}^{m}{\mathcal {Z}}_{t-\tau }(e^s)|_{s=0}\,\hbox {d}\tau . \end{aligned}$$

Hence, using the second statement in Lemma F1, we have

$$\begin{aligned} \frac{{\mathbb {E}}((Y_{t}^{\mathrm {Stoch}})^{m})}{{\mathbb {E}}((Y_{t}^{\mathrm {Det}})^{m})}=\frac{m!}{\lambda ^{m-1}}+O(e^{-\lambda t}) \end{aligned}$$

which is the desired result. $\square$

### Proof of Theorem 6

Immediately from (8), we see

$$\begin{aligned} {\mathbb {P}}(Y_t>xe^{\lambda t}| Y_t>\varepsilon e^{\lambda t})=\frac{{\mathbb {P}}(Y_t>xe^{\lambda t})}{{\mathbb {P}}(Y_t>\varepsilon e^{\lambda t})}=\frac{\int _{0}^{t}n_{t-\tau }{\mathbb {P}}(Z_\tau>xe^{\lambda t})\,\hbox {d}\tau }{\int _{0}^{t}n_{t-\tau }{\mathbb {P}}(Z_\tau >\varepsilon e^{\lambda t})\,\hbox {d}\tau }. \end{aligned}$$

It is enough to examine the numerator. As, from (3),

$$\begin{aligned} {\mathbb {P}}(Z_{t}>k)=(1-\beta {\mathcal {S}}_t^{-1}){\mathcal {S}}_t^{-k}, \quad k\ge 0 \end{aligned}$$

we have

31 $$\begin{aligned} \int _{0}^{t}n_{t-\tau }{\mathbb {P}}(Z_{\tau }>xe^{\lambda t})\,\hbox {d}\tau =\int _{0}^{t}n_{t-\tau }{\mathcal {S}}_{\tau }^{-\lfloor xe^{\lambda t}\rfloor }\,\hbox {d}\tau -\beta \int _{0}^{t}n_{t-\tau }{\mathcal {S}}_{\tau }^{-\lfloor xe^{\lambda t}\rfloor -1}\,\hbox {d}\tau . \end{aligned}$$

Here, $\lfloor a \rfloor$ denotes the integer part of *a* and is necessary as ${\mathbb {P}}(Z_{t}>k)$ is defined on the non-negative integers. Focusing on the first term from the right hand side of (31) and using the definition of ${\mathcal {S}}_{\tau }$ (4) gives

$$\begin{aligned} \int _{0}^{t}n_{t-\tau }\exp \left( -\lfloor x e^{\lambda t} \rfloor (\log (1-\beta e^{-\lambda \tau })-\log (1-e^{-\lambda \tau }))\right) \,\hbox {d}\tau . \end{aligned}$$

Now, we change variables to $s=xe^{\lambda (t-\tau )}$ and note that the resulting integrand can be dominated by $\frac{n_{\lambda ^{-1}\log (s/x)}}{\lambda s}e^{\lambda (1-s)}$ which is integrable for all $n_{\tau }$ under consideration (by Laplace transform arguments, Schiff 1999). Hence, by the dominated convergence theorem, we can conclude

$$\begin{aligned} \lim _{t\rightarrow \infty }\int _{0}^{t}n_{t-\tau }{\mathcal {S}}_{\tau }^{-\lfloor xe^{\lambda t}\rfloor }\,\hbox {d}\tau =\lambda ^{-1}\int _{x}^{\infty }n_{\lambda ^{-1}\log (s/x)}\frac{e^{-\lambda s}}{s}\,\hbox {d}s. \end{aligned}$$

The second integral from the right hand side of (31) can be treated analogously and yields an identical result with $\beta$ as a prefactor. Hence,

$$\begin{aligned} \lim _{t\rightarrow \infty }\int _{0}^{t}n_{t-\tau }{\mathbb {P}}(Z_{\tau }>xe^{\lambda t})\,\hbox {d}\tau =\int _{x}^{\infty }n_{\lambda ^{-1}\log (s/x)}\frac{e^{-\lambda s}}{s}\,\hbox {d}s=\mu ^{-1}m(x,\infty ), \end{aligned}$$

and the claimed result follows. $\square$
